# Food Losses and Waste: A Needed Assessment for Future Policies

**DOI:** 10.3390/ijerph182111586

**Published:** 2021-11-04

**Authors:** Pilar Campoy-Muñoz, Manuel Alejandro Cardenete, María del Carmen Delgado, Ferran Sancho

**Affiliations:** 1 Department of Economics, Universidad Loyola Andalucía, Dos Hermanas, 41704 Seville, Spain; mpcampoy@uloyola.es (P.C.-M.); macardenete@uloyola.es (M.A.C.); 2 Department of Economics, Universidad Autónoma de Barcelona, Bellaterra (Cerdanyola Vallés), 08193 Barcelona, Spain; ferran.sancho@uab.cat

**Keywords:** linear multiplier models, social accounting matrix, food waste, C67, C68

## Abstract

About one third of food produced for human consumption is lost or wasted. For this reason, food losses and waste has become a key priority within worldwide policy circles. This is a major global issue that not only threatens the viability of a sustainable food system but also generates negative externalities in environmental terms. The avoidance of this forbidding wastage would have a positive economic impact on national economies in terms of resource savings. In this paper we look beyond this somewhat traditional resource savings angle and we shift the focus to explore the distributional consequences of food losses and waste reduction using a resource constrained modeling perspective. The impact due to the behavioral shift of each household is therefore explained by two factors. One is the amount of resources saved when the behavioral shift takes place, whereas the other one has to do with the position of households in the food supply chain. By considering the whole supply chain, instead of the common approach based only in reducing waste by consumers, we enrich the empirical knowledge of this issue and improve the quantification of its economic impact. We examine data for three EU countries that present different economic structures (Germany, Spain and Poland) so as to have a broader and more robust viewpoint of the potential results. We find that distributional effects are different for consumers and producers and also across countries. Our results could be useful for policymakers since they indicate that policies should not be driven merely by the size waste but rather on its position within the food supply chain.

## 1. Introduction

Food losses and waste (FLW) means that food itself and all the resources and sink capacities employed along food supply chain (FSC) are wasted. FLW have adverse impacts on environmental and socioeconomic terms with differences between high- and low-income nations [1]. On one hand, FLW negatively impact on landscape and the ecosystem services they provide [2]. FLW also contribute to climate change with GHG emissions [3,4] and have a considerable water and land footprint [1,5,6,7]. On the other hand, FLW implies concerns over food security [8,9] and malnourishment [10,11] and distributive issues of a non-sustainable global food and agriculture system in a context of a growing world population [12]. Thus, FLW and obesity are rising in EU and, simultaneously, 33 million people cannot afford a quality meal every second day making food assistance indispensable in several Member States. Many initiatives have emerged to mitigate FLW [13] but a balance between decrease food waste and requirements of food safety should be also considered in designing such initiatives [14], thus generating a “win-win” situation for both food and nutrition safety and environmental sustainability [1].

FLW occurs at different stages of the FSC, so identifying the most effective points for governance instruments is of the major importance to tackle this problem [15]. Food losses occurs in the earliest stages (primary agriculture and food processing industries that transform agricultural outputs and other inputs to food), being quite frequent in developing economies; whereas food waste is linked to later stages (wholesale and retail sector that distributes the output of the food and the processing industry to the final point of use, that is, households, caterers, canteens, restaurants etc.), prevailing in North America and Europe [12]. Concretely, at the European scale, estimates of FLW range from 158 to 298 kg per capita due to the differences in definitions and accounting methods, but the majority of the studies come to the conclusion that wasting mostly occurs at the consumption stage, especially at households’ level [16,17]. Among them, [18] show that households produce the largest share (42 percent), followed by manufacturing (39 percent), food service/catering (14 percent) and retail/wholesale (5 percent).

FLW has become a key priority within the worldwide policy circles, being particularly pertinent in the light of the Sustainable Development Goals (SDGs) of the UN, adopted in 2015 as part of the Agenda 2030. This includes Goal 2 ‘end hunger’ and Goal 12 ‘responsible consumption and production’. Under the latter, target 12.3 explicitly requires “halving per capita global food waste at the retail and consumer levels by 2030 and reduce food losses along production and supply chains, including post-harvest losses”. In fact, it has been estimated that halving food waste could meet the demand for food of the growing population. To track the progress towards targets 12.3, FAO has developed two indices (Food Loss Index and Food Waste Index). First results have been released in December 2019. However, considerable data challenges in developing these indices still remain [19].

In the same vein, the EU has placed reducing FLW among its top priorities. Already in 2011, the European Commission identified the food sector as key sector in its Roadmap to a Resource Efficient Europe. The reduction of food waste was also a targeted area of the EU action plan for the Circular Economy launched in 2014 [20,21]. Then, the EU committed to UN SDG 12.3 in 2015 and established the EU Platform on Food Losses and Food Waste in 2016 [22], bringing together institutions and private actors to define instruments aimed at preventing waste and the evaluation of their progress. More recently, 2018 revised EU Waste Legislation has called on the EU countries to actively pursue the monitoring and reduction of food waste along the FSC as a contribution to SDG 12.3, being transposed into national legislation by 5 July 2020 [23]. Finally, food waste is of major relevance to achieving sustainability as part of the Farm to Fork Strategy published in May 2020 [24]. In addition, the European Commission has enhanced the consistency and quality of data generated in the EU by two research projects, named Food Use for Social Innovation by Optimising Waste Prevention Strategies (FUSIONS) (2012–2016) [25] and Resource Efficient Food and Drink for the Entire Supply Chain (REFRESH) (2015–2019) [26] and a Delegated Decision (EU) 2019/1597), whose data on FLW are expected to be available by 2022 [12].

Notwithstanding that SDG has put FLW back on to the worldwide political agenda, the evidence to inform policymakers on the magnitude, causes, remedies and impacts of FLW remains extraordinarily sparse [19]. Within this context, this study informs the debate on FLW reduction through a quantitative approach that contribute to the economic analysis of FLW impacts, which are in short supply [27]. [28] offers a global annual monetary estimate of wasted food at USD 2.6 trillion (3.3% of global GDP), of which USD 1 trillion is the direct value of the wasted food, USD 0.9 trillion is linked to the unavoided hunger and the remaining USD 0.7 trillion are due to environmental impacts with GHG emissions and water as the most important items [29]. On the other hand, economic studies about quantitative impact explicitly recognize the direct impacts along FSC, but also the resulting ripple effects for the broader macro-economy. Most of these applied studies employ a Computable General Equilibrium (CGE) approach, a systems-wide macroeconomic simulation approach, focusing on the short-run [30] or the medium-run [31,32] and assessing the impacts in single countries [30,33] or in several regions [32,34,35,36]. Partial Equilibrium (PE) models [37] as well as employing econometric methods [38,39,40]. Some of these studies also clarify the distributional effects of FLW reduction, that is, redistributing wealth/income among different economic agents or regions as purchasing power is a key point in food security issues [15]. Thus, an impact assessment on EU waste management targets also confirms that FLW reductions would benefit manufacturers while food producers and retailers are likely to be worse-off [41]. On the other hand, [20] and [37] found quite different impacts between producers and consumers, but also across countries or regions of the same country.

This work looks further into the distributional consequences of FLW reduction by using a CGE framework. It has the capability of capturing the economic impact of reallocating those resources saved by reducing FLW along the FSC in some EU countries. To do so, we implement budget constrained expenditure multipliers [42] using a Social Accounting Matrix with highly disaggregated agricultural and food industry accounts (Agro-SAMs) for the sample of the three selected Member States (Germany, Spain and Poland), offering a more realistic picture of the wide macroeconomic effects of FLW reductions under different economic structures.

Section 2 presents the methodology, estimates of FLW and the database. Section 3 and Section 4 provide an overview of results and discussion. Section 4 concludes.

## 2. Materials and Methods

### 2.1. The AgroSAM Database

A Social Accounting Matrix (SAM) is the most common reference database for the implementation of the Computable General Equilibrium (CGE) framework, providing data for economic modeling but also a complete and intuitive structural snapshot of the economy under study. The concept of a circular flow of income, underlying a SAM, means that the database reflects the full process of production, trade, income generation and its redistribution between institutional sectors [43]. This allows undertaking a reliable analysis of the distribution of wealth and income, once a savings investment accounts balance and the households’ budget constraint is assumed [44].

The structure of the SAM database is a square matrix in which each account is represented by a row and a column. Transactions are recorded by double entry bookkeeping system of accounting, with income in rows and payment in columns. There are six basic groups of accounts, representative of activities, commodities, production factors and institutional sectors (households and corporations, public institutions, capital accounts and rest of the world). The final dimensions of the matrix are determined by the level of disaggregation of these groups [44].

AgroSAMs is a set of standardized SAMs with disaggregation of the primary sector for each Member State, providing directly comparable structural information for each economy. These follow the same sectoral concordance as the Eurostat Supply and Use Table, with the exception of the agriculture and food accounts drawn from CAPRI database [45]. Thus, of the 97 activity/commodity accounts, 29 cover primary agriculture, one agricultural services sector, seven primary sectors (forestry, fishing and mining activities), 12 food processing, 20 (nonfood) manufacturing and construction, and 29 services sectors. In addition, SAMs contain two production factors (capital and labor), trade and transportation margins and several tax accounts (taxes and subsidies on production and consumption, VAT, import tariffs, direct taxes). Finally, there is a single account for the private household, corporate activities, central government, investments-savings and the rest of the world. The original database is for the year 2000 [46] and then updated to 2007 [47], which is the one employed in this study. From the complete database, SAMs for Spain, Poland and Germany were chosen to perform the proposed analysis, as these countries are representative of different economic structures and different patterns in food purchase and food waste. Considering the composition of their GVA (Gross Value Added), the primary sector is more important in Spain (about 3% of its GVA) and Poland (around 2.5%) than in Germany (0.8%). On the contrary, more than 20% of the GVA is due by industry in Poland and Germany, whilst this percentage drops to 16% in Spain. Services is the most important set of activities, among them “Distributives trades, transport, accommodation and food services” is one of the most important cluster of activities, but its contribution is quite different: Germany is over 15%, Spain is nearly 20% and Poland is close 25% of their corresponding GVA [48]. These differences translate into major difference in wealth. In fact, Germany is one of the wealthy countries, whose GDP per capita in PPS is largely above the EU-27 average, while Spain is near to the said average and Poland is well behind this EU average [49]. Regarding households’ expenditure, “Food and non-alcoholic beverages” is the third most significant item in nearly all EU countries. German households spend below the European average expenditure in this item, whilst Spain is on the average and Poland is quite far away [50]. The order slightly changes when food waste per person is considered: Poland exhibits the fifth position out of 27 member states with 247 kilograms per person, while Germany is holding the 14th position and Spain the 17th, with 149 and 135 kilograms per person, respectively [51].

### 2.2. Estimates of FLW

There are several studies that present estimates of FLW generated at EU level using various data sources, resulting in different figures for wasted food per capita [16]. This ranges from 173 kg/year/per capita of FUSION project or 179 kg/year/capita of [18] to 280–300 kg/year/capita of [52]. The study by [18], commissioned by the European Commission, was the first to present food waste estimates at the Member State level and has therefore become a reference for the EU. Although their figures for different segment of the FSC have been subsequently challenged (e.g., [53]); a number of studies carried out for some member states (e.g., [54]) support the estimates reported by [18].

In this study, the estimates of [18] are employed to determine the wasted food that can be avoided for twofold: food wastage data for EU-27 are provided for year 2006, the closest to the reference year of the AgroSAM database, and disaggregated by the different agents along the FSC, allowing considers the impact due to food losses and waste, with the exception of those happened in agricultural activities or primary sector. According to [18], the Wholesale/Retail0F sector (WRS) (production sector involving the distribution and sale of food products to individuals and organizations)generates the smallest proportion of food waste, only 5%; followed by the Food service/Catering1F sector (FSS) (production sector involved in the preparation of ready-to-eat food for sale to individuals and communities; includes catering and restauration activities in the hospitality industry, schools, hospitals and businesses)which amounts for 14% of the waste. The bulk of food waste is generated by the Manufacturing2F sector (MFS) (production sector involved in the processing and preparation of food products for distribution.) with 39%; but also at Household3F level (HH) (it involves food waste generated at home by consumers in household units) with 42%. From these, the value of the avoidable food waste has been established by applying the corresponding percentage from [18] to the food purchases made by each sector. Concretely, the 6.3% of the food purchases made by WRS and FSS could be avoidable, whereas this figure increases to 15% at HH level. In the FSS, 4% to 10% of food purchases are estimated to become waste before reaching a customer and this waste is 90% avoidable, whereas no data are available from WRS. Due to that, the avoidable portion of food waste for those productive sectors has been calculated by multiplying the midpoint (7%) by the avoidable portion of waste, resulting in the 6.3% of the food purchases. In MFS, food waste is largely unavoidable, so this sector has not been considered in the simulation described just below. Finally, at HH level, food waste represents 25% of food purchases (by weight), of which 60% could be avoidable; therefore, the percentage of avoidable waste has been established in 15%.

Considering the previous information, we put forward four different scenarios to assess the economic impact and the distributional effects of reducing avoidable FLW on the three member states selected:

- Scenario 1: Impact on the member states economies analyzed as a result of reducing the avoidable FLW generated by the overall FSC.
- Scenarios 2 and 3: Impact of reducing the avoidable FLW in WRS and FSS respectively in terms of total output, GDP and employment on the three European economies.
- Scenario 4: Impact resulting of the abatement of the avoidable portion of food which ends up as being discarded by households in terms of total output, GDP and employment on Spanish, German and Polish economies.

### 2.3. Budget-Constrained Multipliers

Based on the multiplier theory initiated by [55] and later expanded by [56,57], budget-constrained multipliers are derived following the same procedure used to calculate the extended multipliers matrix from $Y_{m}={\left(I-A_{mm}\right)}^{-1}\cdot Z$, where *Z* is the vector of exogenous accounts4F $\left(A_{mk}\cdot Y_{k}\right)$ and submatrix *A*_*mk*_ represents how the income flows from the exogenous accounts are distributed among the endogenous accounts).$M={\left(I-A_{mm}\right)}^{-1}$ is the extended multipliers matrix constructed from the SAM. These multipliers can be interpreted as the input requirements by unit increases of expenditure or income (depending on whether columns or rows are considered) in an account, as in the so-called inverse Leontief matrix, with the difference that this matrix reflects the relation between production, the factors’ income, income distribution and final demand. It is important to point out that the selection of *m* (i.e., the decision regarding which accounts are endogenous) usually depends on the type of analysis undertaken, which determines which accounts (exogenous) are the ones explaining the variation of the income in other accounts (endogenous). If changes in the vector of exogenous accounts are denoted as *dZ*, changes in the income of the endogenous accounts will be expressed as

(1) $$dY_{m}=M\cdot dZ=M\cdot d\left(A_{mk}\cdot Y_{k}\right)=M\cdot A_{mk}\cdot dY_{k}$$

The *j^th^* column in *M* indicates the total income generated in each of the endogenous accounts when a unit of income flows from the exogenous institutions towards the *j* endogenous account. Adding up each column in *M* we obtain the standard multipliers $\mu_{j}=\sum_{i=1}^{m}m_{ij}$.

Conversely, if a budget constrain is included in the model, any increase of income to an endogenous account will be followed by a reduction of income to the remaining ones, keeping thus that constraint [42]. This implies the use of a redistribution scheme (*φ*) that guarantees the upholding of the budget constraint of the corresponding agent $\varphi_{j}=\sum_{i\neq j}^{m}\varphi_{i}$ The standard multiplier is now conditioned by the countervailing substitution effects induced by such scheme. As result, we obtain the budget-constrained multiplier matrix $\hat{M}$, where each of its columns can be rewritten as follows:

(2) $${\hat{\mu }}_{j}=\sum_{i=1}^{m}\varphi_{j}\cdot m_{ij}^{}+\sum_{i=1}^{m}\sum_{i\neq j}\varphi_{i}\cdot m_{ij}$$

Unlike the always positive standard multipliers $\mu_{j}$, the budget-constrained multiplier ${\hat{\mu }}_{j}$ can show any sign, either positive or negative, as result of the balance between the overall positive output effects $\left(\sum_{i=1}^{m}\varphi_{j}\cdot m_{ij}\right)$ and the negative substitution effects $\left(\sum_{i=1}^{m}\sum_{i\neq j}\varphi_{i}\cdot m_{ij}\right)$.

In this study, the exogenous vector *Z* is defined for each agent along the supply chain and at household level, encompassing the corresponding demand of agrifood commodities. A new vector Z′ is obtained by subtracting the injection of income resulting of monetizing the avoidable portion of food waste by each agent along the supply chain and at a household level in each member state selected. According to [18], food waste is “waste composed of raw or cooked food materials and includes food materials discarded at any time between farm and fork” whereas, food waste at household level is considered as “waste generated before, during or after food preparation, such as vegetable peelings, meat trimmings, and spoiled or excess ingredients”. In both cases the food waste can be edible or inedible. The economic impact derived from this negative shock described in each scenario is given in absolute terms and in percentage of change over the baseline data encompassed in the corresponding AgroSAM, using both standard and budget-constrained multipliers. Under the latter, while the overall output effect will be negative, the substitution effects will be positive due to the negative shock. It means that the corresponding agent employs the money saved by avoiding food waste in purchasing other goods or services. The redistribution scheme employed for each agent follows a simple homothetic pattern that assumes an increase proportionate to the initial outlays.

## 3. Results

Under the Scenario 1 (Table 1), the Polish economy exhibits the smallest size for the shock (EUR 6868 MM), whereas this shock is nearly double on the Spanish economy (EUR 12,742 MM) and more than four times on the German economy (EUR 29,968 MM). However, in relative terms, countries show a reverse order and the differences among them are not so pronounced under the standard multiplier approach. Thus, the effects on German economy are the smallest, with change on production and GDP of −1.42% and −1.21% respectively. The impact on Spanish economy is slightly higher, with figures of −1.57% and −1.49%, and greater on Polish economy, with a reduction of −2.32% in production and −2.15% in GDP. Employment does not follow the previous pattern since Spain and Poland exhibits lower and quite similar figures in terms of labor shedding compared with Germany, nearly doubling the job lost due to reduce the production of the commodities demanded by WRS, FSS and HH. That picture changes where the same shock is analyzed under the budget-constrained multiplier approach. As expected, the impact of FLW decrease between 50.5% and 66.5% for the variable considered. In addition, while Germany continues to be the economy with the smallest effects and Poland the one with the largest in relative terms, the difference among both countries is lesser. However, the employment pattern remains.

In Scenarios 2 and 3, the portion of avoidable food waste established was the same (6.3% of food purchases) but the monetary size of the shock is quite different for each sector, such as the shock is much smaller within WRS than within FSS. In the case of WRS (Table 2), Germany exhibits the smallest shock (EUR 73 MM) and thus the impact in terms of production and GDP is barely −0.02%, whereas the reduction in labor reaches 6400 employments. These figures are slightly higher for the Spanish economy, where the shock amounts for EUR 108 MM, therefore the production and GDP decrease −0.07% and the labor falls in 11,378 employments. The Polish economy is the most affected by reducing the food waste within the WRS, the shock is EUR 246 MM more than three times the size of the shock in Germany. The impact is also much higher compared to German economy since the production and GDP decreases by −0.33% and the labor falls by 36,580 employments—15 and 16 times the Germany figures. Conversely, when we consider the budget constrained scenario, all the economies turn their figures to positive, that is, the overall output effect is overcame by the substitution effects. This could indicate the promoter capacity of WRS on the corresponding economies.

The size of the shock due to reducing food waste by German and Spanish FSS is much greater than in the corresponding WRS (Table 3). For those countries, the monetary value of the avoidable food waste is over one thousand million Euros. Although the shock in German economy is greater in absolute terms (EUR 1602 M) compared to Spanish economy (EUR 1165 M), the effects on production and GDP are higher for the latter. The same does not apply for labor; labor decreases by 75,989 employments in Germany compared to 54,616 in Spain. It is noteworthy that the smallest shock in Poland, almost nine times less than in Germany, generates a similar impact in terms of production and GDP, but the impact is much less severe on the labor force, with a reduction of 29,915 employments. Although the substitution effects greatly reduce the negative impact of the shock, they are not enough to turn the figures into positive ones, as in the WRS case. This group of activities has lesser influence on the economy compared with WRS.

Scenario 4 reflects the impact of reducing the avoidable food waste generated by HH (Table 4). As pointed out by [18], HH are responsible for the most part of waste arising. Germany exhibits the greatest shock (EUR 28,293 M), Spain is in the midpoint (EUR 11,468 M) and Poland the smallest one (EUR 6434 M). However, the effects in term of production and GDP are quite similar for the first two countries (between −1.36%), whereas they are slightly higher on Polish economy (around −2.48%). Turning attention to labor, the pattern again differs, that is, Germany exhibits the largest reduction of employment, followed at some distance by Poland and in lesser extent by Spain. In this case, the budget-constrained assumption leads to the best situation for Spain, with the highest decrease on the negative impact. Additionally, this time, Poland exhibits better results than Germany. Thus, with a close change in production and GDP after the impact, the effects on employment are quite different.

## 4. Discussion

The use of budget constrained multipliers allows a better understanding of the impact of FLW reduction on the selected economies. The money saved by such reduction is spent on other commodities following the initial pattern of expenditure of each agent. The impact due to the behavioral shift of each agent is explained by the amount of resources (money) saved but also by the position of each agent in the FSC and therefore the relationship of such agent with the remaining ones embodied within the AgroSAM. In this vein, the behavioral shift of household is of major importance as their decrease of demand of food means a reduction in the activity of all the previous agents along the FSC; while the money reallocated to other activities implies an increase in demand that should be meet with a rise in the production of such activities. These results are consistent with the economic theory of food waste and the results stated by [34], where the “*trade-offs occur on the demand side where a reallocation of spending on previously wasted foods causes some producers to be worse off and some to be better off*”. To the best of the authors’ knowledge, there are no further studies considering net effects of household behavioral shift. On the other hand, the impact of household food waste reduction is also analyzed by [32] showing that such reductions lead to falls in EU agrifood production whilst the effect on food price is indeterminate due to the due to the opposing supply and demand forces in food markets. Our study does not capture such effect as prices are fixed in the short run. Furthermore, [32] hypothesized that *investments to reduce food waste by manufactures could generate some efficiency gains from improved packaging and reductions in product losses which may even offer net benefits to those firms that uptake food waste reduction technologies* base on [58]. *Nonetheless, available data does not allow an accurate quantification of these payoffs* [59]. Our time framework also implies that the homothetic pattern employed to reallocate resources is the most appropriate as consumers and producers could not change dramatically their expenditure pattern within this the time interval. In addition, due to the statistical and methodological uncertainty, estimates of avoidable FLW are critical and can vary between studies, leading to different impacts among countries [17,60]. Finally, this study does not include the losses that occurred in the primary sector due to the lack of harmonized data. Regardless of these caveats, our study offers an empirical assessment of the macro-wide effects of FLW reduction showing that distributional effects are different for consumers and producers and also across countries. These outcomes could be used by policymakers as an input to determine what agent of the FSC to focus on and to avoid policies driven merely by the size of waste instead of its impacts.

According to [32], economic modelling representations of food waste reductions in the literature are, by necessity, simplified representations of the multi-layered mechanics which drive household behaviors, as discussed above. Indeed, the focus is more toward the resulting market impacts, which are ultimately influenced by plausible data estimates of food waste, elasticities of demand and supply and the market interactions between consumers and producers [61]. Moreover, the repercussions of reducing household food waste extend beyond the direct impacts on agriculture and food activities, to include the ripple effects on upstream input markets (i.e., feed, fertilizer usage, land and labor), as well as food security benefits arising from reduced food import dependence.

## 5. Conclusions

Food waste reduction lessens the misappropriation of economic resources in a world that faces multiple challenges due to the increasing population growth. Wide economic impact of FLW reduction provides essential policy guidance to tailor interventions that target the prevention and reduction of FLW, including the complete FSC in order to become sustainable while improving food security and nutrition. Considering the whole supply chain instead of focusing only in reducing the waste by consumers, which seems to be the approach taken by most industrialized countries, the policies will have the greatest beneficial impact.

We have used a SAM model in this research since this type of model has several advantages over other methodologies. They are very useful for impact analysis and provide sensible estimates of sectoral and economy wide impacts originating in a change in final demand. They are relatively easy to use and require only a modest amount of training for running the required software. In fact, the hardest question for researchers is the availability of data. When data are available, these methodologies enable the user to quickly conduct certain types of impact analysis. Nevertheless, in regard to further research, the development of a fully Computable General Equilibrium model based as well on a SAM database would provide a better platform for the dynamic representation of economic conditions over the medium and long term. This extension could facilitate a more holistic and complete framework to evaluate the economic impact of food waste reduction. Adaptation to changing market conditions and prices is also of paramount relevance for adequately capturing the behavior of economic agents.

## Figures and Tables

**Table 1:** Economic impact of FLW reduction along FSC (millions of euros, percentage, number of jobs).

MS	Shock	Multiplier Production			GDP		Employment
		Absolute Terms		%	Absolute Terms	%	Absolute Terms %
Germany	−29,968	Standard	−48,563.53	−1.42	−18,134.76	−1.21	−323,528.27
		Constrained	−21,693.98	−0.63	−6188.29	−0.41	−160,075.40
		Differences		−55.3		−65.9	−50.5
Spain	−12,742	Standard	−24,145.97	−1.57	−9,669.65	−1,49	−162,323.37
		Constrained	−12,269.02	−0.80	−4625.99	−0.71	−79,939.60
		Differences		−49.2		−52.2	−50.8
Poland	−6868	Standard	−11,448.09	−2.32	−4185.58	−2.15	−183,380.96
		Constrained	−4496.85	−0.91	−1403.72	−0.72	−74,291.60
		Differences		−60.7		−66.5	−59.5

**Table 2:** Economic impact of FLW reduction by WRS (millions of euros, percentage, number of jobs).

MS	Shock	Multiplier	Production		GDP		Employment	
			Absolute Terms	%	Absolute Terms	%	Absolute Terms	%
Germany	−72.67	Standard	−782.52	−0.02	−370.05	−0.02	−6399.87	
		Constrained	116.54	0.00	65.29	0.00	1100.9	
		Differences		−116.0		−116.4		−117.2
Spain	−108.77	Standard	−1260.62	−0.07	−590.45	−0.07	−11,378.29	
		Constrained	117.58	0.01	57.37	0.01	1195.7	
		Differences		−109.1		−109.1		−110.5
Poland	−246.95	Standard	−1838.02	−0.33	−772.62	−0.32	−36,580.61	
		Constrained	209.54	0.04	108.21	0.05	5030.1	
		Differences		−111.4		−114.1		−113.8

**Table 3:** Economic impact of FLW reduction by FSS (millions of euros, percentage, number of jobs).

MS	Shock	Multiplier	Production		GDP		Employment	
			Absolute Terms	%	Absolute Terms	%	Absolute Terms	%
Germany	−1602	Standard	−10,450.98	−0.28	−4613.71	−0.27	−75,989.38	
		Constrained	−2515.65	−0.07	−1014.76	−0.06	−20,487.30	
		Differences		−75.9		−77.8		−73.0
Spain	−1165	Standard	−6934.86	−0.41	−2994.03	−0,40	−54,615.69	
		Constrained	−1898.40	−0.11	−779.71	−0.11	−14,955.00	
		Differences		−72.7	−	74.0		−72.6
Poland	−187	Standard	−1684.07	−0.30	−690.20	−0.30	−29,915.10	
		Constrained	−233.74	−0.04	−88.78	−0.04	−4828.70	
		Differences		−86.0		−87.2		−83.9

**Table 4:** Economic impact of FLW reduction by HH (millions of euros, percentage, number of jobs).

MS	Shock	Multiplier Production			GDP		Employment	
		Absolute Terms		%	Absolute Terms	%	Absolute Terms	%
Germany	−28,293	Standard	−68,688.45	−1.52	−29,647.68	−1.36	−576,486.73	
		Constrained	−20,225.75	−0.45	−5737.21	−0.26	−146,757.10	
		Differences		−70.6		−80.6		−74.5
Spain	−11,468	Standard	−30,548.80	−1.47	−13,569.28	−1.36	−259,869.57	
		Constrained	−7672.94	−0.37	−2,439.78	−0.24	−33,844.30	
		Differences		−74.9		−82.0		−87.0
Poland	−6434	Standard	−16,763.42	−2.65	−6958.15	−2.48	−332,327.08	
		Constrained	−4391.56	−0.69	−1393.38	−0.50	−66,493.70	
		Differences		−73.8		−80.0		−80.0
